# Quality and Physicochemical Traits of Carcasses and Meat from Geese Fed with Lupin-Rich Feed

**DOI:** 10.3390/ani10030519

**Published:** 2020-03-20

**Authors:** Joanna Kuźniacka, Marcin Hejdysz, Mirosław Banaszak, Jakub Biesek, Sebastian Kaczmarek, Małgorzata Grabowicz, Andrzej Rutkowski, Marek Adamski

**Affiliations:** 1 Department of Animal Breeding, Faculty of Animal Breeding and Biology, UTP - University of Science and Technology in Bydgoszcz, Mazowiecka 28, 85-084 Bydgoszcz, Poland; kuzniacka@utp.edu.pl (J.K.); miroslaw.banaszak@utp.edu.pl (M.B.); adamski@utp.edu.pl (M.A.); 2 Department of Animal Breeding and Product Quality Assessment, UP Poznań University of Life Sciences, Wołyńska 33, 60-637 Poznań, Poland; marcin.hejdysz@up.poznan.pl; 3 Department of Animal Nutrition and Feed Management, Poznań University of Life Sciences, Wołyńska 33, 60-637 Poznań, Poland; sebastian.kaczmarek@up.poznan.pl (S.K.); marhej@up.poznan.pl (A.R.); 4 Department of Physiology, Zoophysiotherapy and Animal Nutrition, Faculty of Animal Breeding and Biology, UTP - University of Science and Technology in Bydgoszcz, UTP - University of Science and Technology in Bydgoszcz, Mazowiecka 28, 85-084 Bydgoszcz, Poland; malgorzata.grabowicz@utp.edu.pl

**Keywords:** carcass, fatness, goose, lupin, muscles, meat quality, protein

## Abstract

**Simple Summary:**

In the past, lupin seeds were not used in poultry feeding because of their alkaloid content, which affected the growth performance, producing negative results. Nowadays, new cultivars of lupins have reduced the anti-nutrition content and have been characterized by a high level of protein, similar to the level in soybean meal. Goose production is not popular and, generally, should be done by an extensive or semi-intensive system, which is corelated with small costs of feeds. Lupins can be used for crops where it is not possible to produce soybean (environmental conditions). Our study indicated that it is possible to produce goose meat with a good quality, where the feeding was based on various cultivars of lupins. Yellow, as well as white, lupin-rich feed had a positive effect on the meat traits and meat quality. Narrow-leaved lupin worsens the growth performance.

**Abstract:**

The aim of the study was to analyze the quality of geese meat receiving feed with soybean meal (group 1), yellow lupin (group 2), narrow-leaved lupin (group 3), or white lupin (group 4). In total, 400 male White Kołuda^®^ geese were randomly assigned to four groups, with 10 replicates and 10 birds each, during the 77-day rearing period. After the end of the rearing period, 10 birds from each group were slaughtered and dissected. Meat quality traits were measured. Based on the production results, it can be concluded that geese use fodder with yellow and white lupin to the same degree as in the case of the control group and higher body weight gain was recorded in the first rearing period. In contrast, the use of narrow-leaved lupin in mixtures for geese worsened the feed used. Meat traits were similar in all groups, including the content of muscles and fat in the carcass (*p* > 0.05), excluding abdominal fat. The weight of abdominal fat and its proportion in the carcass were higher (*p* < 0.05) in geese from group 4. A higher (*p* < 0.05) pH was found in group 1. The protein and intramuscular fat content in breast muscles was highest (*p* < 0.05) in geese from group 4, and a higher water content was found in group 1. The protein content in leg muscles was higher in group 3, and the fat content was higher in group 4 (*p* < 0.05). The color and water-holding capacity of meat were comparable in all groups (*p* > 0.05). The analysis revealed a positive effect of replacing soybean meal with alternative protein sources, especially yellow and white lupin, on the growth performance and quality of goose meat.

## 1. Introduction

Most broiler geese produced in Poland (95%) are two-strain crossbreeds designated W31 (White Kołuda Geese^®^). They are reared for 13 weeks, and for the last 3 weeks before slaughter, fattened on oats. There is a possibility to rear younger geese for slaughter, which includes 10–11 weeks without fattening by oats [1,2,3]. Meat quality is an important issue for potential consumers. The quality assessment of meat products is based on physical traits. For consumers, the color of meat and its water-holding capacity play important roles. These traits determine the suitability of raw meat for further processing. They also influence the economic aspect of meat production. The quality of meat from broiler geese depends on the genotype, age of birds, and their management system, particularly the diet. The quality and quantity of muscle tissue in the carcass of birds depend on the composition of feed used in their diet. The diet of young oat geese (commercial: White Kołuda^®^ geese) should contain 22.0–15.0% of crude protein and 12–11.7 MJ of metabolizable energy (ME) [3,4,5,6]. These geese are a crossbreed (W31) of two strains (W11 × W33) and characterized by a good quality of meat and fat in carcasses, which adds to the flavor of meat [1]. Soybean meal (SBM) is a popular protein-rich component of feed used in poultry diets. It is usually sourced from genetically modified organisms (GMO). Interest in alternative sources of vegetable protein has been increasing because of the need to secure the supply of protein for agricultural production in Poland [7,8,9]. Lupin seeds are a good source of protein, as well as dietary fiber and other nutritional components [10]. High-protein sources such as yellow lupin (*Lupinus luteus* L.), narrow-leaved lupin (*Lupinus angustifolius*), or white lupin (*Lupinus albus*) can be used in poultry diets. Lupins contain up to 40% protein. New cultivars of lupin contain reduced levels of alkaloids and other antinutrients. In the past, high levels of these compounds created problems with the use of lupins in poultry diets because they reduced the palatability and intake of feed [11,12,13,14,15]. Nowadays, lupin seeds have a lower concentration of alkaloids than in the past, but the other antinutritional factors are still present in seeds, and the contents of alkaloids and raffinose depend on the species and variety of lupins [16]. The content of alkaloids or non-starch-polysaccharides (NSP) could have an unbeneficial effect on energy utilization and even diarrhea [17]. NSP may have an impact on growth performance. Additionally, raffinose and phytate-phosphorus are from antinutritional substances that could negatively affect poultry production [18] The content of antinutritional factors depends on the cultivar and growing season of plants. This was discussed by Kaczmarek et al. (2014), where the nutritional value of narrow-leaved lupin was studied. Chiofalo et al. [19] reported that lupin seeds are interesting material to use in animal feed. The quality of diets could be improved by using lupin seeds. In the study of Biesek et al. [20], geese were fed with soybean meal, as a control, and with yellow lupin seeds with the addition of potato protein, and brewers’ yeast. The authors obtained the final product (meat after fattening by oats) and concluded that it could be used as a protein source in goose diets, especially on small-scale farms, with their own crop resources. Haraf [21] reviewed the research, and dealt with the impact of goose nutrition and the genotype on carcass characteristics and meat quality. The review discussed how nutrition affects the quality of meat and modifying the composition of feed can give producers different results for the final product. It was mentioned in the review that the replacement of soybean meal with other protein components (e.g., sunflower meal and yellow lupin seeds) may affect the modification of the fatty acid profile in goose meat, as recommended by dietitians. According to Hejdysz et al. [18], the use of white or yellow lupin in chicken diets resulted in good growth performance parameters. This was in line with the research of Smulikowska et al. [22], as well as Olver and Jonker [23], who recommended this lupin species.

The tested hypothesis is as follows: Lupin seeds of various cultivars used as a high-protein component as a substitute for soybean meal in complete feed have an effect on the quality of meat from geese.

The aim of the present study was to analyze the quality of meat from broiler geese receiving balanced feed containing protein sourced from yellow lupin, narrow-leaved lupin, white lupin, potato protein, and brewers’ yeast as substitutes for soybean meal.

## 2. Materials and Methods

This study aimed to examine meat quality. It is part of a large multiannual project (information in the Acknowledgement section) on the use of protein sources from vegetables in monogastric animal feeding. The research material was obtained after professional slaughter. All research methods and procedures were conducted according to the relevant European Union (EU) directives and the protocol for this study was approved by the Local Ethics and Animal Experimentation Committee at Poznan University of Life Science, and followed all the requirements in relation to animal welfare.

### 2.1. Lupin Seeds

Table 1 presents the chemical composition and value of the seeds of the narrow-leaved lupine, Sonet cv.; yellow lupin, Mister cv.; and white lupin, Butan cv. (commercial cultivars). Seeds were obtained from two plant breeding stations in Przebędowo and Wiatrowo (Poland). A hammer mill, model RG11 (Zuptor, Gostyń, Poland), was used to ground the seeds. A 2.0 mm screen was used.

### 2.2. Animals and Diets

The study was conducted on 400 males of White Kołuda^®^ geese, divided into four groups, with 10 replicates and 10 birds per group. Geese were reared for 77 days in pens (3 × 2 m) on litter and with a limited range (5 × 3 m), according to the commonly used technology of goose rearing. Geese were kept in pens where the temperature was 22–24 °C (at the beginning, an additional source of heat was provided, resulting in a temperature of 32 °C). The first 3 weeks’ light duration was 24 h, and then 16 h per day. Group 1 (control) received balanced feed containing soybean meal (SBM), and group 2 (experimental) received balanced feed containing yellow lupin (*Lupinus luteus* L.). Group 3 (experminetal) was fed a diet based on narrow-leaved lupin (*Lupinus angustifolius*), and group 4 (experminetal) was fed a diet based on white lupin (*Lupinus albus)*. Lupins were in ground form. Feed was in the form of a complate granulated mix. The diet of birds from groups 2, 3, and 4 also contained protein sourced from potato and brewers’ yeast. There were two feeding phases. On days 1–42 of rearing, the birds received feed containing 50% of protein concentrate with minerals, and they then received feed with 40% of protein concentrate with minerals on days 43–77. The second component of the diet (50–60%) was triticale. The composition of feed and concentrate is presented in Table 2 and Table 3. Values are based on the recommended allowances and nutritive value of feedstuffs [25]. Feed and concentrates were obtained by using the program HYBRIMIN WinFumi 7.

### 2.3. Growth Performance

In the study, the body weight gain (BWG), feed intake (FI), and feed conversion ratio (FCR) were calcuted. Each parameter was checked for three periods, separately, that is, 0–42 d, 43–77 d, and for the whole rearing period of 0–77 d.

### 2.4. Chemical Analyses

Using a 0.5 mm sieve, lupin seeds and experimental diets were ground as representative samples. The analysis was done in two repetitions for dry matter, crude protein, and ether extract by methods 943.01, 976.05, and 920.39, respectively [26]. The level of starch in experimental seeds was determined with the diagnostic assay kit (Megazyme International; AOAC 2005; 2007, method 996.11). The neutral detergent fiber (aNDF-NDF, with heat-stable amylase and expressed inclusive of residual ash, method 973.18) was determined. The AA content was determined on an AAA-400 Automatic Amino Acid Analyzer (INGOS s.r.o., Prague, Czech Republic), using ninhydrin for postcolumn derivatization (procedure 994.12; AOAC [26]). In this study, alkaloids were analyzed via gas chromatography (GC) (Shimadzu GC17A, Kyoto, Japan) using a capillary column (Phenomenex, Torrance, CA, USA). Raffinose, stachyose, and verbascose were the oligosaccharides. These substances were determined by high-resolution gas chromatography, as well as the phytate content of lupin meals. The content of NSP was analyzed by gas–liquid chromatography (neutral sugars) and colorimetry (uronic acids), with modifications. The viscosity of the water extract of lupin seeds was measured in vitro by the method described by Kaczmarek et al. [27]. The chemical analyses of lupin seeds and diets used in our study were similar to the methods described in the research of Kuźniacka et al. [28].

### 2.5. Meat Quality

After the end of the rearing period (77th day), 10 birds from each group were slaughtered (according to the regulations applied in the poultry industry). Birds were chosen randomly. Birds were without feed on the slaughter day. The slaughtering procedure was done by cutting the head (rapid bleeding), after prior stunning with an electric current. Plucked and gutted carcasses, excluded head and feet were analyzed for qualitative parameters. All analyses of meat quality were conducted one day after the slaughter (24 h). The pH value of breast muscles was measured 15 min post-mortem (pH_15_). Carcasses were placed in cold storage (2 °C; 24 h) and the pH was measured again (pH_24_) using a CX-701 pH-meter with a knife electrode (Elmetron, Zabrze, Poland). The test of pH was done at a depth of 2.5 cm below the surface of breast muscles. The carcasses were weighed on RADWAG scales with an accuracy to the nearest 0.01 g (Radwag, Radom, Poland). Carcasses were dissected [29], and the following parts were separated: Breast muscles; leg muscles; skin with subcutaneous fat (included legs’ skin); abdominal fat; offal (liver, heart, and gizzard); wings with skin; neck with skin; and total carcass remains, including the trunk and leg (femur and hip) bones. Each carcass element was weighed. Whole breast and leg muscles (without separating parts) were used in the other analysis. The color of breast (*pectoralis major*) and leg (*biceps femoris*) muscles was analyzed with a colorimeter (Konica Minolta, model CR400, Tokyo, Japan), calibrated using the white calibration plate no. 21033065 and the D_65_Y_86.1_x_0.3188_y_0.3362_ scale. The color was graded according to the International Commission on Illumination’ system for L* (lightness), a* (redness), and b* (yellowness) [30]. The color was measured on the surface of the muscles. Color was measured for each breast and leg muscle from group once (10 measurements per group). Drip loss from breast muscles was measured. Breast muscles were weighed post-mortem, suspended in transparent bags with holes for water leakage, and after 24-h storage at 2 °C, were weighted again [31]. The water-holding capacity of breast and leg muscles was analyzed using a method by Grau and Hamm [32]. Pooled samples of homogenized muscles (0.300 g ± 5%) were wrapped in Whatman grade 1 filter paper and kept under a 2 kg pressure for 5 min. There were 10 repetitons of analysis per group for breast and leg muscles. The water-holding capacity of meat was calculated based on the difference in weight before and after the test. Pooled samples of homogenized breast and leg muscles, without separating tendons and intramusclur fat (90 g), from each study group were analyzed in a laboratory for the content of protein, collagen, salt, connective tissue, fat, and water, according to the PN-A-82109:2010 [33] standard with the FoodScan apparatus from FOSS (Warsaw, Poland), using the Near InfraRed Transmission (NIT) spectrometer calibrated for the artificial neural network (ANN).

### 2.6. Statistical Analysis

Numerical data were analyzed using statistical software STATISTICA 10.0 PL (2011). Mean values of the examined parameters were calculated using one-way analysis of variance (ANOVA). The pooled standard error of the mean (SEM) was also calculated. The significance of differences between groups was verified with the post-hoc Scheffe test at the significance level of *p* < 0.05. Differences between groups were considered significant when the *p*-value was <0.05.

## 3. Results and Discussion

### 3.1. Chemical Composition of Lupin Seeds

Lupin seeds were characterized by the different contents of protein and the other substances. The most protein-rich seeds were found in yellow lupin seeds (over 421 g/kg DM), whereas the ether extract in this cultivar was the smallest level (55.1 g/kg DM), without statistical differences (*p* > 0.05). The results of neutral detergent fiber, total oligosaccharides (RFO), and the phytate P showed that yellow lupin seeds had the highest content of these substances, which was a negative trait. Generally, there were no significant differences between lupin species that were used in our study. In another study, the authors [34] reported that lupin seeds are a good alternative to substitute soybean meal. They also showed that yellow lupin is the most protein-rich plant, compared to narrow-leaved and white lupin, which was confirmed in our research. Kuźniacka et al. [28] also used yellow and narrow-leaved lupin. They reported a higher content of protein in narrow-leaved lupin (30.3%) than in our study, but the protein in yellow lupin seeds was at a similar level. Differences could also be found in the NDF content. In the study of Rutkowski et al. [35], the NDF content was higher in narrow-leaved and yellow lupin seeds than in our research. It could have been affected by the other crops that were used. Musco et al. [36] studied the chemical composition of three lupin species, which were *Lupins albus, Lupinus luteus*, and *Lupinus angustifolius.* They showed that the differences within one species can be very high, depending on the variety that was used. However, all of the cited authors mentioned that lupin seeds are rich in protein and are a promising replacement to soybean. Nowadays, consumers need a wider choice on the agri-food market. There are also many expectations in terms of limiting genetically modified products, and in many countries, it is not possible to produce soybean meal due to the prevailing conditions. Lupins could be an alternative and their cultivation on poultry meat farms will allow producers to be self-sufficient.

### 3.2. Growth Performance

The mortality of geese was very low and not more than 1%. In the first rearing period (0–42 d), the highest body weight gain (BWG) in groups of geese fed with yellow lupin (4.054 kg) and white lupin (4.023 kg) was found in comparison to the control group, where the soybean meal was the protein source (3.806 kg), *p <* 0.0001. In the second period of rearing (43–77 d), as well as in the whole rearing period (0–77 d), no statically significant differences were found between groups (*p* > 0.05). In the same groups (2 and 4), feed intake was also higher than in the control group (1) in the first period, but in the second period (43–77 d), geese fed with narrow-leaved lupin were characterized by a higher feed intake than other groups (*p* < 0.05). Finally, during the whole rearing period, treatment groups had a higher feed intake (more less by 0.85–1.32 kg) than the control group (*p* < 0.05). The feed conversion ratio (FCR) in the first period was the highest in the 3rd group, where geese were fed with feed based on narrow-leaved lupin (2.40 kg/kg), than in the other groups. Too high FCR is a negative trait, because geese need to intake more for 1 kg of body weight gain. The smallest FCR, without statistical confirmation (*p* > 0.05), was in the group of geese fed with yellow lupin (2.21 kg/kg). At the end of the rearing period, the FCR paramter was also the highest in the 3rd group (3.30 kg/kg). The worst growth performance in the 4th group, where geese were fed with narrow-leaved lupin, was noted (Table 4). The reason for decreased growth performance could be due to the chemical composition of lupin seeds. In Table 1, a high concentration of soluble NSP in narrow-leaved lupin seeds was found compared to the other lupin species (*p* > 0.05). As Kaczmarek et al. (27) reported, non-starch polysaccharide (NSP) contents negatively affect the growth and feed intake. This was confirmed by Hejdysz et al. [37]. In another study, yellow lupin in the diets of 10-week-old geese did not affect the body weight gain [38]. Similarly, Laudadio and Tufarelli [39] did not find a negative effect of white lupin on broiler chickens’ growth performace. Smulikowska et al. [22] reported lower performance of chickens fed with narrow-leaved and yellow lupin, but those with narrow-leaved-rich diets had the worst FCR (*p* < 0.05). Mikulski et al. [40] concluded that narrow-leaved lupin in turkeys’ diets could be used, without a negative effect on the growth performance. The differences between our results and the other studies could be due to the use of different species of birds and various cultivars of lupins, and there could have been an effect of maintaince conditions or production technology.

### 3.3. Carcass Traits

The analysis of meat traits in broiler geese did not show significant differences between groups, *p* > 0.05 (Table 5). The pre-slaughter body weight was highest in birds fed white lupin (group 4). Birds from the control group (1) were 186.67 g lighter than in group 4, but 333.33 g heavier than birds fed narrow-leaved lupin (group 3). Differences in the carcass weight were similar. The dressing percentage was highest, above 63%, in groups 1 and 4, without statistical confirmation. It was related to the carcass weight and muscle weight, which are related to the live body weight of each bird, FI, and FCR. This result was more than 2% higher than in group 3. The weight of offal in group 2 was 3.96 g lower than in group 1. The weight of carcass remains was highest in groups 2 and 4 (over 1 kg). Groups did not differ significantly (*p* > 0.05) for the weight of muscles (Table 6). Bieliński et al. [41] fed Italian White geese a diet with the inclusion of sweet lupin at a level of 12–15% per feed ration. After a 10-week rearing period, they found no negative effect of the tested feed on the performance parameters of geese, weight gain, or egg laying. A study by Biesiada-Drzazga et al. [38] investigated the effects of yellow lupin (25–50%) as a substitute for soybean meal in a goose diet. The study revealed that a diet containing yellow lupin did not deteriorate the weight of the carcass or breast and leg muscles from 10-week-old birds. The weight of the carcass and content of muscles in our study also showed no differences when lupins were included in the diet. However, the highest weight and proportion of breast muscles in the carcass were found in group 2, but without statistical confirmation (*p* > 0.05). The weight of leg muscles, total weight of muscles, and their proportion in the carcass were slightly higher in group 1 (*p >* 0.05). The weight of abdominal fat and its proportion in the carcass were significantly higher (*p* < 0.05) in group 4 compared to group 1. The total content of fat in the carcass was lowest in group 3, but the differences with other groups were not significant (Table 6). Biesiada-Drzazga [42] also tested the effect of yellow lupin and sunflower meal as a partial replacement of SBM and reported that geese fed this diet had a reduced weight of breast muscles and significantly lower content of fat in the carcass. On the other hand, our study found a significantly higher content of abdominal fat in geese fed white lupin. The content of fat in the carcass of geese fed different lupin species was also higher than in geese fed the SBM diet, although the differences were not significant (*p >* 0.05). This could be due to the other (higher) level of fat (ether extract) in seeds of lupins than in soybean meal. A 10% dietary inclusion of yellow lupin per feed ration for White Kołuda geese^®^ was also tested by Pietrzak et al. [43]. Their study showed, similar to our findings, no negative effects of yellow lupin on the quality parameters of the carcass or meat. The diet with the inclusion of white lupin increased the content of fat in breast and leg muscles. Similarly, Biesek et al. [20] did not find a negative effect on most carcass traits, excluding leg muscles and their percentage content in carcasses. However, the values of the obtained results in the cited research were higher. Probably, there was an effect of fattening by oats (the technology of fattening by oats casues more fat in goose carcasses) and age (longer rearing period, until 16 weeks) of geese when slaughtered.

### 3.4. Physicochemical Traits

The value of the pH for breast muscles measured 24 h post-mortem was significantly higher in group 1 (*p* < 0.05) than in group 4 (Table 7). This could have been caused by the slaughter process and related to stress. However, the reduced pH (pH_15_ to pH_24_) indicated normal glycolysis in the muscle tissue after slaughter. The color and water-holding capacity of breast and leg muscles did not differ significantly (*p* > 0.05). The drip loss measured in our study in all groups was in the range of 0.92–0.98% (*p* > 0.05). The juiciness of muscles expressed as the value of drip loss was comparable in all analyzed groups. Augustyńska-Prejsnar and Sokołowicz [44] concluded that a lower value of drip loss indicates a higher juiciness of meat. The color of the meat depends on myoglobin. Its concentration in muscles depends on its physiological activity and the type of muscle fibers [45]. A higher myoglobin content is found in muscle containing fibers that oxidize more slowly [46]. The ability to retain water (drip loss) may depend on the mechanism associated with protein markers [47]. Meat that loses less water is characterized by suitability for further technological processing and has a significant impact on the texture of meat [48]. The lack of significant differences in lightness (L*), redness (a*), and yellowness (b*) in the breast muscles and leg muscles in our own research indicates no differences in the structure of goose muscles in each group. Biesek et al. [20] showed that the color of breast and leg muscles, as well as the chemical composition of muscles, were similar to our results. It could indicate that legumes have the same effect, or that the genotype of geese, which is White Kołuda^®^ geese, gave the described results. The lower ability in terms of the water-holding capacity in the muscle tissue may be due to an increase in the space between the fibers due to the muscle and the sarcomere shortening process of rigor mortis. A lack of significant differences in the ability of water retention between groups indicates that the sarcomere shortening process was similar in each group. The meat aging degradation of cytoskeletal proteins results in slackening of the sarcomere structure and may allow the meat to retain water [49]. The contents of protein, salt, and fat were significantly higher in group 4 than in the other groups. The content of connective tissue was significantly higher in groups 1 and 3, while group 3 was characterized by a significantly higher content of collagen. The significantly highest content of water was found in breast muscles from group 1. Group 3 was characterized by a significantly higher content of protein in leg muscles. The content of collagen and connective tissue was higher in groups 1 and 3. The content of salt and fat was higher in group 4. The significantly highest (*p* < 0.05) content of water was found in leg muscles from groups 2 and 3 (Table 7 and Table 8). According to Tyra and Mitka [50], a higher content of fat, which is the carrier of flavors, may improve the palatability of meat. Similarly, Gumułka and Połtowicz [51] obtained a protein content in muscles of White Kołuda^®^ geese of over 21% and 20%, a little bit more than in our research. It could be affected by differences in bird conditions or nutrition. The collagen and connective tissue plays a main role in the tenderness of meat. If the content is too high, meat can be characterized by a lower tenderness and juiciness. It also depends on the age of birds [52], but in our study, birds were the same age. According to Jamroz and Kubizna [53,54], the level of lupins in feed mixture for geese should be 5–10% for growing birds and 10–15% for adult birds.

## 4. Conclusions

To conclude, the proposed feed rations containing 34.49% of yellow lupin, 34.2% of narrow-leaved lupin, and 35% of white lupin can be used as substitutes for diets with a 32.5% inclusion of soybean meal. The study demonstrated that the inclusion of lupins in a goose diet had a positive effect on the quality of meat. The use of narrow-leaved lupin had no negative effect on the meat traits, but worsened the growth performance. The obtained results indicate that this type of rearing with the use of yellow and white lupin as a subsitute to soybean had no negative effect and the next stage, which is fattening by oats, could be done. More studies on this subject should be conducted.

## Figures and Tables

**Table 1:** Chemical composition of seeds of three lupin species (g/kg DM) ^1^.

Component	Narrow-Leaved Lupin	Yellow Lupin	White Lupin
Dry matter	887	885	886
Crude protein	261	421	351
Ether extract	57.2	55.1	109
Starch	9.02	10.6	11.1
Neutral detergent fiber	218	253	224
Total oligosaccharides (RFO ^2^)	60.0	112	80.7
Raffinose	12.3	10.9	7.21
Stachyose	28.3	63.3	64.2
Verbascose	19.4	37.5	9.3
Phytate P	6.20	7.82	5.10
Alkaloids	0.42	0.41	0.18
Total NSP ^3^	401	317	292
Soluble NSP ^4^	176.1	58.5	66.1
Simple sugars ^5^	10.4	14.6	12.8
Viscosity (cP) ^6^	2.14	1.63	1.55
Amino acids (g/100g of crude protein)			
Asp	9.01	9.51	10.31
Thr	3.13	2.85	3.63
Ser	4.47	4.51	4.85
Glu	20.7	21.3	19.51
Pro	4.11	3.34	3.95
Gly	3.64	3.62	3.84
Ala	3.04	2.98	3.28
Met + cys	1.61	2.52	2.09
Val	3.47	3.23	3.92
Ile	3.63	3.67	4.15
Leu	6.42	7.12	7.01
Tyr	3.33	2.48	4.34
Phe	3.67	3.66	4.21
Lys	4.27	4.52	4.62
His	2.64	2.78	2.47
Arg	10.31	11.62	10.44

^1^ Each value represents the mean of two replicates. ^2^ RFO, raffinose family oligosaccharides. ^3^ NSP, non-starch polysaccharides. ^4^ Soluble NSP were calculated as the difference between NSP and NDF (neutral detergent fiber) [24]. ^5^ Includes glucose and fructose. ^6^ Water extract viscosity. Value based on the recommended allowances and nutritive value of feedstuffs [25]. Value was calculated in an in vivo experiment.

**Table 2:** Composition of feed used during a 77-day rearing period of broiler geese.

Days 1–42	Group ^1^			
	1	2	3	4
Triticale, %	50	50	50	50
Concentrates, %	50	50	50	50
Calculated nutritional value of feed				
Metabolizable energy (ME), MJ/kg	11.55	11.54	11.53	11.53
Crude protein, %	20.52	20.51	20.44	20.47
Calcium, %	1.00	1.00	1.00	1.00
*p*-available, %	0.40	0.40	0.41	0.41
Lysine, %	1.17	1.10	1.10	1.10
Methionine, %	0.41	0.41	0.41	0.41
Valine, %	0.89	0.80	1.00	0.97
Threonine, %	0.81	0.81	0.81	0.81
Na, %	0.16	0.16	0.16	0.16
Cl, %	0.14	0.14	0.14	0.14
Days 43–77	1	2	3	4
Triticale, %	60	60	60	60
Concentrates, %	40	40	40	40
Calculated nutritional value of concentrates				
Metabolizable energy (ME), MJ/kg	11.65	11.67	11.64	11.66
Crude protein, %	18.01	18.03	18.00	18.01
Calcium, %	0.81	0.81	0.81	0.81
*p*-available, %	0.36	0.36	0.36	0.36
Lysine, %	0.90	0.90	0.90	0.90
Methionine, %	0.45	0.45	0.45	0.45
Valine, %	0.73	0.73	0.73	0.73
Threonine, %	0.70	0.70	0.70	0.70
Na, %	0.16	0.16	0.16	0.16
Cl, %	0.14	0.14	0.14	0.14

^1^ 1, control group fed with soybean meal; 2, experimental group fed with yellow lupin; 3, experimental group fed with narrow-leaved lupin; 4, experimental group fed with white lupin.

**Table 3:** Composition of concentrates used during a 77-day rearing period of broiler geese.

Composition of concentrates, %	Group ^1^			
	1	2	3	4
Soybean meal, 44%	65	-	-	-
Yellow lupin	-	68.98	-	-
Narrow-leaved lupin	-	-	68.4	-
White lupin	-	-	-	70
Potato protein	-	3	8	6
Brewers’ yeast	-	3	8	6
Triticale in concentrate	23.04	12	1.22	2
Soybean oil	5.2	5.4	7.6	8.8
Premix ^2^	2	2	2	2
Fodder chalk	2	2	1.92	1.58
Monocalcium phosphate	1.52	1.74	1.54	2.16
NaHCO_3_	0.84	0.8	0.8	0.8
Fodder salt	0.18	0.12	0.14	0.14
L-lysine	-	0.32	0.08	0.14
DL-methionine	0.2	0.4	0.28	0.32
L-threonine	0.02	0.24	-	0.02
Tryptophan	-	-	0.02	0.04
Calculated nutritional value of concentrates				
Metabolizable energy (ME), MJ/kg	10.79	10.79	10.77	10.79
Crude protein, %	31.42	31.43	31.44	31.47
Calcium, %	1.92	1.92	1.92	1.92
*p*-available, %	0.56	0.56	0.56	0.56
Lysine, %	1.82	1.82	1.82	1.82
Methionine, %	0.65	0.65	0.65	0.65
Valine, %	1.14	1.14	1.14	1.14
Threonine, %	1.28	1.28	1.28	1.28
Na, %	0.31	0.31	0.31	0.31
Cl, %	0.19	0.19	0.19	0.19

^1^ 1, control group fed with soybean meal; 2, experimental group fed with yellow lupin; 3, experimental group fed with narrow-leaved lupin; 4, experimental group fed with white lupin. ^2^ The compound premix provides per kg of diet: Cu, 10 mg; Fe, 60 mg; Mn, 80 mg; Zn, 60 mg; I, 1.5 mg; Se, 0.3 mg; vitamin A, 10.000 IU; vitamin D, 2500 IU; vitamin E, 25 IU; vitamin K, 1.0 mg; vitamin B1, 2.0 mg; vitamin B2, 8.0 mg; vitamin B6, 2.5 mg; vitamin B12, 0.01 mg; vitamin PP (nicotinamide pancreatic polypeptide), 30.0 mg; vitamin B5, 15.0 mg; vitamin B9, 0.5 mg; and biotin, 0.15 mg.

**Table 4:** Productivity paramters of geese during the rearing period.

Group	BWG 0–42	BWG 43–77	BWG 0–77	FI 0–42	FI 43–77	FI 0–77	FCR 0–42	FCR 43–77	FCR 0–77
1	3.81 ^b^	2.74	6.64	8.53 ^b^	11.81 ^b^	20.34 ^b^	2.25 ^b^	4.38	3.07 ^b^
2	4.05 ^a^	2.72	6.87	8.99 ^a^	12.41 ^b^	21.40 ^a^	2.21 ^b^	4.61	3.12 ^b^
3	3.54 ^c^	2.94	6.58	8.48 ^b^	13.18 ^a^	21.66 ^a^	2.40 ^a^	4.55	3.30 ^a^
4	4.02 ^a^	2.91	6.88	9.09 ^a^	12.10 ^b^	21.19 ^a^	2.26 ^b^	4.25	3.08 ^b^
SEM	0.04	0.06	0.06	0.06	0.15	0.15	0.02	0.08	0.03
*p*	<0.0001	0.457	0.144	<0.0001	0.003	0.005	0.005	0.411	0.029

^a, b^, mean values marked in columns with different letters are significantly different between groups, *p* < 0.05; 1, control group fed with soybean meal; 2, experimental group fed with yellow lupin; 3, experimental group fed with narrow-leaved lupin; 4, experimental group fed with white lupin; days: 0–42, 43–77, and 0–77. BWG, body weight gain (kg); FI, feed intake (kg); FCR, feed convertion ratio (kg/kg).

**Table 5:** Meat traits of 77-day-old broiler geese *.

Group ^1^	Pre-Slaughter Body Weight (g)	Weight of Carcass (g)	Dressing Percentage (%)	Weight and Proportion in Carcass				Weight of Offal (g)	Carcass Remains (g)
				Neck with Skin		Wings			
				g	%	g	%		
1	6743	4262	63.26	348.0	8.13	675.0	15.87	369.1	9054
2	6910	4318	62.48	272.0	6.38	638.0	14.73	365.2	1128
3	6410	3894	60.99	285.4	7.30	562.6	14.40	325.9	967.1
4	6930	4385	63.37	346.3	7.95	632.5	14.51	335.9	1001
SEM	135.08	87.70	0.71	18.25	3.76	26.34	3.45	13.70	56.06
*p*-value	0.525	0.197	0.648	0.247	0.410	0.101	0.163	0.332	0.371

* No significant differences, *p*-value > 0.05; ^1^ 1, control group fed with soybean meal; 2, experimental group fed with yellow lupin; 3, experimental group fed with narrow-leaved lupin; 4, experimental group fed with white lupin.

**Table 6:** Content of muscles and fat in 77-day-old broiler geese.

Group ^1^	Weight and Proportion in Carcass											
	Breast Muscles		Leg Muscles		Total Muscles		Skin with Subcutaneous Fat		Abdominal Fat		Total Fat	
	g	%	g	%	g	%	g	%	G	%	g	%
1	606.6	14.23	642.9	15.10	1250	29.33	1086	25.51	135.2 ^b^	3.16 ^b^	1222	18.17
2	650.9	14.98	575.9	13.51	1227	28.48	970.6	22.43	185.5 ^a,b^	4.39 ^a,b^	1156	16.77
3	547.8	14.23	562.6	14.52	1111	28.75	901.4	23.06	176.0 ^a,b^	4.46 ^a,b^	1077	16.72
4	583.2	13.32	621.0	14.27	1204	27.60	1127	25.70	223.1 ^a^	5.08 ^a^	1350	19.61
SEM	26.78	3.49	24.10	3.48	49.41	2.94	49.53	3.12	11.06	3.88	58.58	3.37
*p*-value	0.272	0.448	0.140	0.520	0.172	0.697	0.068	0.080	0.033	0.038	0.096	0.165

^a, b^, mean values marked in columns with different letters are significantly different between groups, *p* < 0.05; ^1^ 1, control group fed with soybean meal; 2, experimental group fed with yellow lupin; 3, experimental group fed with narrow-leaved lupin; 4, experimental group fed with white lupin.

**Table 7:** Physicochemical parameters of breast muscles from 77-day-old broiler geese.

Group ^1^	pH_15_	pH_24_	Color ^2^			Water-Holding Capacity (%)	Drip Loss (%)	Protein (%)	Collagen (%)	Salt (%)	Connective Tissue (%)	Fat (%)	Water (%)
			L*	a*	B*								
1	8.17	7.98 ^a^	38.97	18.29	3.16	39.50	0.98	20.82 ^d^	1.40 ^a,b^	0.53 ^b^	6.71 ^b^	3.16 ^c^	74.71 ^a^
2	7.85	7.79 ^a,b^	36.98	19.45	4.06	36.56	0.92	20.59 ^c^	1.27 ^a,b^	0.58 ^b^	6.15 ^c^	3.64 ^b^	73.89 ^d^
3	7.97	7.89 ^a,b^	39.30	17.60	3.22	36.72	0.96	21.01 ^b^	1.53 ^a^	0.60 ^b^	7.28 ^a^	3.19 ^c^	74.20 ^b^
4	8.02	7.71 ^b^	40.27	17.48	3.03	36.09	0.95	21.77 ^a^	1.16 ^b^	1.07 ^a^	5.32 ^d^	3.90 ^a^	72.47 ^c^
SEM	3.72	3.73	2.55	3.34	3.92	2.73	4.00	3.21	3.99	4.02	3.78	3.91	1.06
*p*-value	0.065	0.002	0.274	0.342	0.617	0.643	0.992	<0.0001	0.002	<0.0001	0.004	<0.0001	<0.0001

^a, b^, mean values marked in columns with different letters are significantly different between groups, *p* < 0.05; ^1^ 1, control group fed with soybean meal; 2, experimental group fed with yellow lupin; 3, experimental group fed with narrow-leaved lupin; 4, experimental group fed with white lupin; ^2^ L*, lightness; a*, redness; b*, yellowness.

**Table 8:** Physicochemical parameters of leg muscles from 77-day-old broiler geese.

Group ^1^	Color ^2^			Water-Holding Capacity (%)	Protein (%)	Collagen (%)	Salt (%)	Connective Tissue (%)	Fat (%)	Water (%)
	L*	a*	b*							
1	42.26	14.28	4.23	34.57	19.03 ^c^	1.57 ^a,b^	0.53 ^c^	8.24 ^a^	7.23 ^b^	71.95 ^b^
2	42.97	13.86	4.11	35.11	19.53 ^b^	1.39 ^c^	0.55 ^c^	7.11 ^c^	5.72 ^c^	73.09 ^a^
3	43.06	15.69	5.22	34.64	19.99 ^a^	1.62 ^a^	0.61 ^b^	8.12 ^a,b^	5.39 ^d^	73.06 ^a^
4	40.85	15.30	4.26	38.67	18.88 ^d^	1.46 ^b,c^	0.94 ^a^	7.73 ^b^	8.92 ^a^	70.13 ^c^
SEM	2.41	3.48	3.88	2.75	3.26	3.97	4.02	3.73	3.82	1.13
*p*-value	0.474	0.512	0.679	0.342	<0.0001	0.002	<0.0001	0.001	<0.0001	<0.0001

^a, b^, mean values marked in columns with different letters are significantly different between groups, *p* < 0.05; ^1^ 1, control group fed with soybean meal; 2, experimental group fed with yellow lupin; 3, experimental group fed with narrow-leaved lupin; 4, experimental group fed with white lupin; ^2^ L*, lightness; a*, redness; b*, yellowness.
